# Clinicians’ use of Health Information Exchange technologies for medication reconciliation in the U.S. Department of Veterans Affairs: a qualitative analysis

**DOI:** 10.1186/s12913-024-11690-w

**Published:** 2024-10-08

**Authors:** Margie E. Snyder, Khoa A. Nguyen, Himalaya Patel, Steven L. Sanchez, Morgan Traylor, Michelle J. Robinson, Teresa M. Damush, Peter Taber, Amanda S. Mixon, Vincent S. Fan, April Savoy, Rachel A. Dismore, Brian W. Porter, Kenneth S. Boockvar, David A. Haggstrom, Emily R. Locke, Bryan S. Gibson, Susan H. Byerly, Michael Weiner, Alissa L. Russ-Jara

**Affiliations:** 1 College of Pharmacy, Purdue University, West Lafayette, IN USA; 2 Center for Health Information and Communication, Health Services Research and Development Service CIN 13-416, U.S. Department of Veterans Affairs (VA), Veterans Health Administration, Richard L. Roudebush VA Medical Center, 1481 West 10th Street (11H), Indianapolis, IN 46202 USA; 3 Department of Medicine, Indiana University School of Medicine, Indianapolis, IN USA; 4 Center for Health Services Research, Regenstrief Institute, Inc, Indianapolis, IN USA; 5 College of Pharmacy, University of Florida, Gainesville, FL USA; 6 Information, Decision Enhancement and Analytics Center of Innovation, U.S. Department of Veteran Affairs, Veteran Health Administration, Salt Lake City, UT USA; 7 Department of Biomedical Informatics, Decision Enhancement and Analytics Sciences 2.0 Center of Innovation VA, University of Utah, Salt Lake City, UT USA; 8 Geriatric Research, Education, and Clinical Center, VA Tennessee Valley Healthcare System, Nashville, TN USA; 9 School of Medicine, Vanderbilt University Medical Center, Nashville, TN USA; 10 VA Puget Sound Health Care System, Seattle, WA USA; 11 School of Medicine, University of Washington, Seattle, WA USA; 12 Purdue School of Engineering and Technology, Indiana University-Purdue University Indianapolis, Indianapolis, IN USA; 13 University of Tennessee Health Sciences Center, Memphis, TN USA; 14 Birmingham VA Medical Center, Geriatrics Research Education & Clinical Center, Birmingham, AL USA; 15 Division of Gerontology, Geriatrics and Palliative Care, University of Alabama at Birmingham, Birmingham, AL USA; 16 Department of Biomedical Informatics, School of Medicine, University of Utah, Salt Lake City, UT USA; 17 Regenstrief Center for Healthcare Engineering, Purdue University, West Lafayette, IN USA

**Keywords:** Health Information Exchange, Medical Informatics, Health Information Technology, Medication Reconciliation, Safety, Patient

## Abstract

**Background:**

Medication reconciliation is essential for optimizing medication use. In part to promote effective medication reconciliation, the Department of Veterans Affairs (VA) invested substantial resources in health information exchange (HIE) technologies. The objectives of this qualitative study were to characterize VA clinicians’ use of HIE tools for medication reconciliation in their clinical practice and to identify facilitators and barriers.

**Methods:**

We recruited inpatient and outpatient prescribers (physicians, nurse practitioners, physician assistants) and pharmacists at four geographically distinct VA medical centers for observations and interviews. Participants were observed as they interacted with HIE or medication reconciliation tools during routine work. Participants were interviewed about clinical decision-making pertaining to medication reconciliation and use of HIE tools, and about barriers and facilitators to use of the tools. Qualitative data were analyzed via inductive and deductive approaches using a priori codes.

**Results:**

A total of 63 clinicians participated. Over half (58%) were female, and the mean duration of VA clinical experience was 7 (range 0–32) years. *Underlying motivators* for clinicians seeking data external to their VA medical center were having new patients, current patients receiving care from an external institution, and clinicians’ concerns about possible medication discrepancies among institutions. *Facilitators* for using HIE software were clinicians’ familiarity with the HIE software, clinicians’ belief that medication information would be available within HIE, and their confidence in the ability to find HIE medication-related data of interest quickly. Six overarching *barriers* to HIE software use for medication coordination included visual clutter and information overload within the HIE display; challenges with HIE interface navigation; lack of integration between HIE and other electronic health record interfaces, necessitating multiple logins and application switching; concerns with the dependability of HIE medication information; unfamiliarity with HIE tools; and a lack of HIE data from non-VA facilities.

**Conclusions:**

This study is believed to be the first to qualitatively characterize clinicians’ HIE use with respect to medication reconciliation. Results inform recommendations to optimize HIE use for medication management activities. We expect that healthcare organizations and software vendors will be able to apply the findings to develop more effective and usable HIE information displays.

**Supplementary Information:**

The online version contains supplementary material available at 10.1186/s12913-024-11690-w.

## Introduction

The majority (80%) of U.S. Veterans are “dual” users: they receive care from both the U.S. Department of Veterans Affairs (VA) and a non-VA institution [1]. These individuals require special attention to coordinate care across multiple institutions, a need also emphasized by the Maintaining Internal Systems and Strengthening Integrated Outside Networks Act of 2018 [2]. One important component of clinicians’ care coordination is medication reconciliation. This activity is intended to identify and resolve medication discrepancies, and involves several activities- including comparing all medications prescribed or being taken- to identify duplications, omissions, dosing errors, and conflicting medications. Clinicians are expected to reconcile medications at all major points in care, including hospital admission and subsequent outpatient follow-up visits. A reconciled medication record is then intended to be shared with the patient and the care team. Two VA studies showed that, for most participating patients, at least one medication discrepancy was identified, revealing problems with the accuracy of medication information in medical records [3, 4].

In part, to promote effective medication reconciliation and other aspects of medical care, healthcare organizations such as the VA have invested substantial resources in health information exchange (HIE) technologies. These technologies draw from multiple electronic health record (EHR) systems to aggregate data (including medication data) across multiple healthcare organizations. This information supplements the patient’s EHR data from a single organization. HIE is expected to help clinicians prevent adverse drug events (ADEs) for patients, identify conflicting medications, and generate a more accurate and complete medication record for the patient. Unfortunately, HIE technologies remain underutilized by clinicians [1, 5, 6]. In the VA setting, clinician adoption of HIE technologies has been a focus since 2016, but as of March 2019, only 17% of VA staff with HIE access were active users of HIE [1]. Moreover, a 2018 review of five years of HIE use in the VA found that clinicians reviewed external data only 20% of the time when accessing patient charts from 2013 to 2018 [7]. Clinicians’ motivations for HIE data use, as well as facilitators and barriers to HIE tool use, remain unknown [8]. Therefore, the objectives of this study were to conduct an observational study to qualitatively characterize clinicians’ use of HIE tools for medication reconciliation in their clinical practice, and to determine specific facilitators and barriers to clinicians’ use of HIE tools for medication reconciliation activities.

## Methods

### Study design

This research was conducted in the VA healthcare system, which has facilities across the U.S. We conducted a qualitative investigation using direct observation of clinicians during clinical care, along with semi-structured interviews with individual clinicians. Observations and interviews occurred at four geographically dispersed VA medical centers (VAMC).

### Study setting and HIE technologies

The VA has a large role in promoting HIE in the US [7]. After enabling HIE among its own facilities and with the US Department of Defense (DoD), the VA has promoted HIE partnerships with non-government “community” healthcare institutions starting in 2014, an effort known first as the Veterans Lifetime Electronic Record [9] and later as the Veterans Health Information Exchange (VHIE) [7]. As of this writing, the VA participates in one- or two-way HIE with over 250 partners across the nation [10], increasing the quantity of HIE records available to VA clinicians.

VA clinicians have several ways to access HIE records. During the study, VA clinicians could use any of three internally developed software applications: Remote Data View [11, 12], VistA Web [13], and Joint Longitudinal Viewer (JLV) [14]. Remote Data View provides records to clinicians from other VA sites and DoD as preformatted reports in Computerized Patient Record System, which used to be the primary graphical user interface to VA’s EHR. VistA Web and JLV were web-based tools offering data from VAMCs, the DoD, and VHIE partners [15]. Although VistA Web was still in use during this study, it was later being replaced by JLV, which offers a more modern user interface. The VA continues to maintain all three tools during the study.

### Conceptual framework

This research was guided by the System Engineering Initiative for Patient Safety (SEIPS) framework [16]. This framework facilitates descriptions of how various factors of the work system (technology and tools, physical environment, organization, persons including clinicians and patients) influence clinicians’ processes, which in turn influence clinical outcomes. SEIPS was chosen because this investigation is focused on understanding the role of *technology* (i.e., HIE tools) in supporting clinician *task* (medication reconciliation) completion. Specifically, clinicians collect and compare medication lists from varying sources to identify potential errors. These *processes* are expected to improve *outcomes*.

### Clinician participants

Our goal for sampling was 16 clinicians from each of the four VAMCs (ideally 8 inpatient and 8 outpatient) for a total of 64 participants. Eligible clinicians included those who conducted any activity related to medication reconciliation for inpatient admission or associated outpatient follow-up visits. We specifically sought prescribers (physicians, nurse practitioners, and physician assistants) and pharmacists. Students, residents (house staff), and fellows were excluded, as were clinicians known to be off work or practicing offsite during the interviewers’ visit. Study personnel at each site identified potential participants by obtaining staff directories from the site’s administration team.

For recruitment, clinicians were contacted in-person, via secure instant messaging (e.g., Microsoft Skype for Business) or via encrypted e-mail. In recognition of clinicians’ time to participate, a medication management book valued at $20 was offered to each clinician participant at sites that permitted compensation. Study procedures were approved by the Indiana University Institutional Review Board and the VAMC Research and Development Committees at each study site.

### Data collection

Data were collected between August 2018 and February 2019. Clinicians self-reported demographic data to provide further context for qualitative data, including: practice setting, whether trained in VA, years employed by VA, percent of time in clinic, race, age, and gender.

To conduct observations, two to three members of the research team who had experience in medicine or pharmacy, direct observation, rapid ethnography, or human-computer interaction traveled to each site to collect data over a period of approximately three days per site. Team members separately shadowed participating clinicians during business hours, scheduled in half-day increments, as clinicians interacted with HIE or medication reconciliation tools during their routine clinical work. Clinician-patient interactions were observed only if both the clinician and patient verbally agreed to be observed after reviewing a study information sheet. De-identified observable activities and verbalizations were recorded via handwritten notes and captured on a standardized paper form that was developed by the larger study team. Each researcher then typed their notes into an electronic format for analysis.

For interviews, a draft interview guide was developed by an interdisciplinary subset of the study team with experience in qualitative research and pilot tested with several other members of the research team. Final interview questions (Additional File 1) were designed to elicit in-depth discussion about clinicians’ decision-making pertaining to medication reconciliation activities and use of HIE tools, as well as specific barriers and facilitators to clinicians’ use of VA HIE tools for medication reconciliation activities.

Interviews were generally conducted in-person at the end of each observation period, or during natural breaks in clinical activities to avoid disrupting clinician workflow. At the request of participants, interviews could be scheduled on a different day as a follow-up to observations. Interviews were scheduled for up to 60 min and were audio-recorded with the permission of participants. Recordings were professionally transcribed for analysis.

### Data analysis

Participating clinician demographics were summarized using descriptive statistics computed with Excel 2016 (Microsoft, Redmond, Washington.)

A hybrid (i.e., both deductive and inductive process) was used to analyze the qualitative observation and interview data and to identify motivations for HIE use and its barriers or facilitators. We applied the SEIPS constructs in the development of a priori deductive codes [16–18]. Codes were updated inductively as analysis continued. Specifically, four team members independently coded a total of six transcripts (both interviews and observation notes) to refine the initial codebook following consensus discussion of coding. The coding team members’ expertise spanned human factors engineering, public health, and pharmacy. Coders without prior qualitative analysis experience were trained by senior team members who had qualitative expertise. After this initial refinement of the codebook, the four team members independently coded each of the remaining transcripts (both interviews and observation notes.) Independent coding was performed manually in Word 2016 (Microsoft, Redmond, Washington); consensus codes were assigned in NVivo 12 (QSR International, Melbourne, Australia) to facilitate data quality checks and further analysis of data across clinician demographics. Specifically, to ensure consistency across coders and fidelity to the codebook definitions, a fifth team member with expertise in pharmacy and qualitative methods reviewed coding reports from NVivo and met with the coding team to discuss any adjustments each week. In addition, the full set of transcripts (both interviews and observation notes) from a subset (*n* = 8) of participants was also coded independently by the same four team members, with the fifth member reviewing as a quality check. After initial coding was completed, sub-codes were identified and applied to the transcripts to elucidate findings more fully [19]. Following all coding, the full analysis team met to confirm final codes applied to the findings.

Potential differences in findings by demographic variables were also explored. Specifically, we examined crosstabs (coding frequencies) and framework matrices (quotations from transcripts) in NVivo for any differences by clinician type (physician, other prescriber [i.e., nurse practitioner or physician assistant) pharmacist], site location, and clinicians’ VA work experience (0–4, 5–9, 10–14, 15 + years). Findings pertaining to clinicians’ HIE-related decision-making, including facilitators and barriers to the use of HIE technologies, were mapped to the SEIPS framework. Reporting for this research followed the Consolidated Criteria for Reporting Qualitative Research checklist [20].

## Results

### Participant demographics

A total of 66 clinicians participated in one or more components of data collection; 63 provided demographic information (Table 1). At the request of clinicians, ten interviews (18%) were scheduled on a different day as a follow-up to observations. Three of these were conducted by telephone.

**Table 1:** Characteristics of clinical staff participating in the study

Characteristics	Physicians	Pharmacists	Other Prescribers^b^	Total
Total number of participants^a^	38	17	8	63
Participated in observation^c^	33	17	8	58
Participated in interview^c^	33	17	7	57
Gender, female, n,( %)	15 (43)	13 (76)	7 (88)	35 (58)
Trained in VA, n (%)	32 (91)	12 (71)	6 (75)	50 (83)
Years employed by VA, n (%)				
0–4 years	12 (34)	7 (41)	1 (13)	20 (33)
5–9 years	11 (31)	3 (18)	0	14 (23)
10–14 years	5 (14)	4 (24)	5 (62)	14 (23)
15 + years	7 (20)	3 (18)	2 (25)	12 (20)
Percentage of professional time in clinical activity (Mean and SD)	65 ± 29	81 ± 21	78 ± 19	71 ± 28
Inpatient provider only, n (%)^d^	5 (14)	7 (41)	3 (38%)	15 (25)
Outpatient provider only, n (%)^d^	20 (57)	6 (35)	5 (63%)	31 (52)
Inpatient and outpatient provider, n (%)^c^	10 (28)	4 (24)	0	14 (23)
Race and ethnicity, n (%)				
White	28 (80)	12 (71)	6 (75)	46 (77)
Asian	5 (8)	4 (24)	0	9 (15)
Black	1 (2)	1 (6)	1 (13)	3 (5)
Hawaiian or Pacific Islander	1 (2)	0	0	1 (2)
Hispanic or Latino	0	0	1 (13)	1 (2)
Age, years, n (%)				
20–30	4 (11)	3 (19)	0	7 (12)
31–40	11 (31)	9 (56)	3 (38)	23 (39)
41–50	13 (37)	3 (19)	2 (25)	18 (31)
51+	7 (20)	1 (6)	3 (38)	11 (19)

^a^Total includes 16 participants at Site A, 15 at Site B, 14 at Site C, and 18 at Site D

^b^Group consists of 7 nurse practitioners and one physician’s assistant

^c^Demographic data were missing for 3 physicians

^d^Participants reporting ≥ 80% of their time in either an inpatient or outpatient setting were included in the “inpatient” or “outpatient” category accordingly. Participants reporting ratios less than 80/20 were included in the “inpatient and outpatient” category

### Emergent findings

**Fig. 1:**
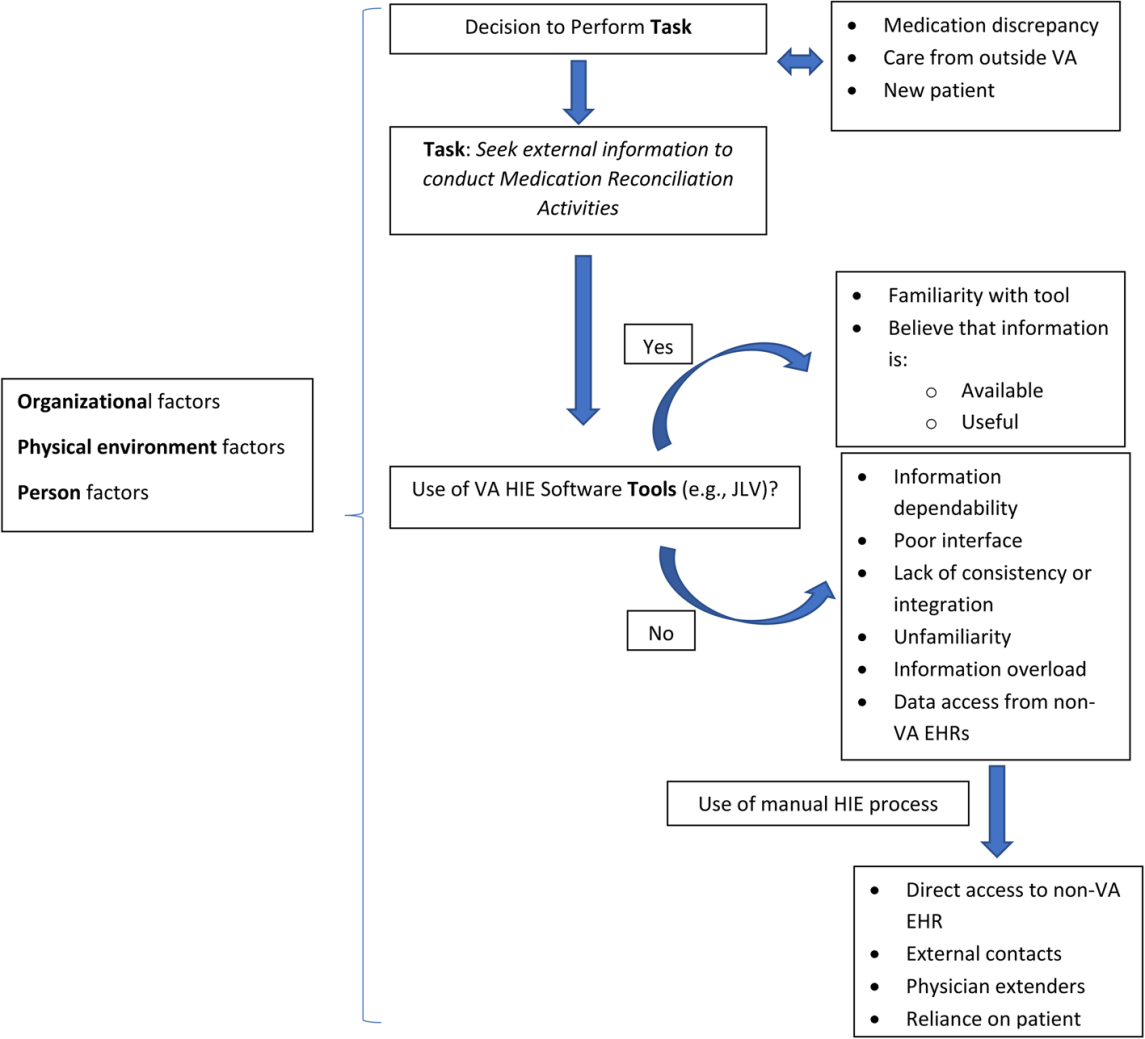
Emergent findings for decision-making, barriers, and facilitators mapped to SEIPS constructs

Findings for the *task* of medication reconciliation and associated HIE use are organized by SEIPS constructs, indicated in *italics* (Fig. 1). No noteworthy differences in findings were observed when comparing across clinician type, study site location, or clinicians’ years of work experience. Illustrative clinician quotations are provided below, with the site and clinician type denoted in brackets (Table 2).

**Table 2:** Salient quotes from clinician participants

Example Quote	Speaker and Site	Themes	SEIPS Construct
“If the patient doesn’t know their home medicines, if there’s a discrepancy, if there’s a medicine that doesn’t seem reasonable based on our information or doesn’t have a reason [medication indication] based on our information, and often times that’s in the geriatric population, a patient that’s confused or… being admitted to the hospital with altered mental status. Those are kind of the key times that I would use [VistA Web].”	[Physician; Site C]	Motivations for use of HIE tools	Medication discrepancy
“With [our clinic], it’s mainly patients who are on allergy injections and they’ll see an outside allergist, so it’s not perfect, but I will review the outside [medication] records to see if something different may have been started by an outside clinician. Those records aren’t consistently in the chart but that’s basically through VistA.”	[Physician; Site C]		Care from outside VA
“So primarily we use CPRS. JLV or VistA Web is mostly for patients who transfer their care from an outside source, and who will say, ‘I’m not sure about my medication,’ or ‘I take this medication,’ that way it helps us to go outside of CPRS into that particular VA and find out the list. That’s the only situation, and mostly with transfer patients or brand-new patients who come from outside. *Those patients [that] are established already in the [our] system and the information is there in CPRS*,* so Vista Web or JLV may not be important in that aspect.”*	[Physician; Site C]		New patients
“I think it’s [VistA Web] organized fine. I don’t really see any issue with it from… the way it looks. Usually, we’ve got a couple different screens. On the left-hand side is some of their old information like H&Ps and progress notes, and then on the right side they’ll pop up the images and those actual notes. That works fine for me.”	[Physician; Site C]	Facilitators of use of HIE tools	Familiarity with tool
“VistA Web is faster than JLV, and I’m more familiar with it, and it seems to get me the information I need in a – more quickly and in a format that I’m used to, so I still prefer it, but I guess we’re all gonna have to go to JLV. But generally, I think it’s arranged chronologically and you have to choose, do I want the past three months, six months, one year, or all information, so you can sort of define a date range but it’s not a custom [range]. I don’t think you can do a custom date range…You just kind of have your preselected options and you can select from those.”	[Physician; Site A]		Familiarity with tool
“Yeah. I mean they [VistA Web and JLV] do *provide useful information*,* and I do rely on them*.”	[Physician; Site B]		Believe information is useful
“The med rec [tool specific to one region] helps though because it actually, in one place, pools all of that information for you to view so you don’t have to go to VistA Web and the CPRS, and this place and to this place. It sort of just puts all of it in one place so that when you want to use the information it’s where you need it.”	[Physician; Site B]		Believe information is available
“I think that it [JLV] works. When the documents are available, it’s fine. I don’t have a problem with it.”	[Nurse Practitioner; Site D]		Believe information is available
“*There almost appears to be too much information in there and it can be kind of hard to sort through*.”	[Physician; Site C]	Barriers to use of HIE tools	Information overload
“*Interfacing is most important. They don’t interface* [i.e., integrate] them. CPRS is one, JLV is another one. I think you can access JLV through CPRS, but if a lot of systems could interface in private sector it would be nice. Interfacing is one. Also, the other person, if they don’t update it then you’re gonna be dependent on that particular clinician [who saw the patient] before you, so *there is a human element of making sure medications are updated*. That’s why the information from the patient would be crucial, and if I update it from the patient from JLV from all our available sources and I make a good updated medication list, then it will continue like that. So *there’s an effort from the doctor*,* effort from the patient*,* making sure the system is also – all systems are designed to be the way they’re built for and they have flaws in their system*….”	[Physician; Site C]		Inadequate interface
“It’s [i.e., VistA Web] onerous to use. Like the subcategories are hard to navigate and you may be looking for something and it may not be in the category you think it’s in, so it just seems to take a long time.”	[Physician; Site C]		Information overload
“I’m not sure what that system is actually called. I’m just starting to use it actually because it’s fairly new for me… I think that *the big gap is when veterans receive non-VA care and what you do then*,* and I think that that’s a little more challenging*.”	[Physician; Site B]		Unfamiliarity
“I think that the biggest problem that I have is that I have to log into another interface. So, I don’t like that. *That just takes time*,* and I wish that it was seamless*.”	[Physician; Site B]		Lack of consistency or integration
“The JLV interface *I don’t find particularly user-friendly*,* and so it’s slow*, and it takes me a long time to extract information that I want… VistA, again, it’s kind of slow going to find the notes that are going to be useful, and they’re often kind of buried, and they again take time to identify and pull up. So, I think that those are the main barriers for those systems.”	[Physician; Site D]		Inadequate interface
“Yeah, the biggest, the issues that we have with HIE would be, and recently what we have found is rather than using like VLER or other systems to get records ported in, it’s becoming more effective to get guest accounts, because now more and more [EHRs] are letting you get read-only access, so I can go to large hospital systems in the area that I know we share a lot of patients with and I can just get a read-only access to their [commercial EHR system], and then I can just log into their system and pull down the records. That’s actually; *it seems to work better than health information exchanges*,* which is frustrating and depressing*.”	[Physician; Site D]	Use of manual HIE process	Direct access to non-VA EHR
“A lot of times, since we’re right next door, a lot of our patients will get part of their care here at the VA but also part of their care at [external healthcare organization] here, so I’ll either, while I’m on over here, I’ll log in virtually into that system or I’ll just simply walk over there and log into the system and look at records that way as well, too.”	[Physician; Site C]		External contacts
“The challenge is if they’re admitted to a private facility and they don’t bring in their discharge summaries or discharge paperwork, you know. Sometimes I do call outside pharmacies if they had it filled like at [supermarket pharmacy] or [mass merchandiser pharmacy], I call them and…then make a note.”	[Physician; Site C]		Physician extenders
“For a new patient, there might be more requests for records. Sometimes we ask our clinic staff to try to get records from another facility to either fax them over or sometimes patients bring them on paper, so it’s a combination.”	[Physician; Site A]		Physician extenders
“I have to depend on getting the outside records so that’s when I ask our nurse to …contact that facility, get a release of information.”	[Physician; Site A]		Physician extenders
“So I usually ask patients that receive medications to bring in their pills, so that I can go through them with the patient or…. That doesn’t happen very often that people are willing to do that, but to have them bring in handwritten lists, and if there is something that is skeptical or confusing, often times I will have the pharmacist help me to do a med rec or call the patient’s outside pharmacy to clarify what they’ve actually received.”	[Physician; Site A]		Reliance on patient
“The [medical] residents have access to data that comes from [Regional Health Network] care as well. So often, *the fact that we have residents in clinic with us is helpful for pulling together records from multiple systems*, and they’re essentially just logging in remotely to their electronic medical records to try to gather data. So that happens fairly frequently.”	[Physician; Site A]		Physician extenders
“A lot of times, since we’re right next door, a lot of our patients will get part of their care here at the VA but also part of their care at the [University Hospital] here, so I’ll either, while I’m on over here, I’ll log in virtually into that system or I’ll just simply walk over there and log into the system and look at records that way as well too.”	[Physician; Site C]	Physical environment level factors	Proximity among facilities
“Like one of the things that I’ve found most helpful is patient positioning while you’re doing a visit. I usually introduce the computer when I’m meeting somebody. Hey, you know, we’re gonna work together as a team, let’s build your medical record so it’s the most accurate possible, and I use pretty much the same phrasing with each patient, and then I position them right there so they can see us working on it [EHR] together and try to check the accuracy.”	[Physician; Site B]		Work rooms
“But the other problem is that throughout the VA, if you ….don’t have the printer registered with your profile, then you’ve got to go and find the printer, and so since I work in different areas of the hospital…. if I see a patient like up on the floor and I want to print their med rec for them, I have to make sure that whatever computer that I’m at has the 8-South printer registered to me so that I can print to it. There are several different computers throughout the 8-South, and on each one, you have to go through and register that printer to your profile, which is a big barrier to printing anything. So it’s not just like you can just go in and search for a printer real quick and then hit print. You’ve got to like actually load it into your profile. So it’s frustrating.”	[Nurse Practitioner; Site B]		Printer availability
“*It would be nice if we had more flexibility and more patient rooms.* In private practice, physicians usually have two rooms and a clinic nurse, dedicated clinic nurse, who will triage the patients, see the patients, see the patients for preliminary interview, fill all the refills and renewals that will be needed, send them to us for signature, get a history from the patient as to what clinical events have been recently. *So that’s a luxury we don’t have at [our facility] where we have a single room and exam*,* so it limits the maximum number of patients we can see in a clinic session having only one room*,* and all we’re provided with really is the vital signs for the patients*. It’s up to us to review the chart and work out the details of what’s been done before or if there’s something different about the patient that needs particular attention.”	[Physician; Site C]		People
“I don’t know that I’ve ever had any training for JLV.”	[Physician; Site A]	Organization level factors	Staffing and training
“*I really don’t feel like I’ve had much training exposure to how to most effectively use JLV*.”	[Physician; Site D]		Staffing and training
“We rely heavily on our pharmacists here to help us with some of that data as well. We’re really lucky to have that here … If I have questions about sort of their home medications, I’ll usually ask them first because they’re more experienced with using the system and able to really sort of sort out, you know, if this was truly a home medicine or if they, and/or if they were, you know, the patient was getting it refilled and how often and things like that, so…”	[Physician; Site C]		Dedicated medications reconciliation staff
“As a hospitalist, a lot of times our job is we want to be able to give them the proper treatment for their acute issue without really doing, in general without doing too much changing to their outpatient medication regimen. That’s my personal practice, unless it’s that outpatient medication regimen that’s causing the problem. Then I’ll intervene.”	[Physician; Site C]	Person level factors	Practice philosophies
“In a rheumatology clinic appointment, we typically focus on rheumatology medications or those medications which interact with their medications, which aren’t so many actually. I always see med rec on the meds I’m prescribing like looking at refill frequency and if other medications are expired… *[I] won’t necessarily do a comprehensive medication reconciliation of everything*,* unless there’s a specific concern brought to me by a patient*.”	[Physician; Site D]		Time available
“Well, a lot of times, they don’t know or they won’t know what they’re taking, but the family, if they come with them, will. But we have a lot of patients with cognitive and memory impairment. So, it does make it difficult when they come alone.”	[Nurse Practitioner; Site D]		Lack of patient familiarity with their medications
“Talking to the patient. They’re the only ones that can tell you what they’re taking. This [HIE] list can show you what’s active. *You can see the refill history*,* but someone can pick up their medications and not take them. So that doesn’t tell you everything. You need to talk to the patient or a family member*, friend, or anyone who knows anything.”	[Pharmacist; Site A]		Clinician-patient communication
“On Vet Link [onsite patient-facing kiosk] … I know that my patients use it to log in and look at results of imaging and labs, and some patients use it to refill their medications. But I wonder if there would be a way for the patients to submit an alert for like a new medication or something. Like they went to urgent care, and now they have this antibiotic, and they could put it in there themselves or at least notify us that something has changed. The way that I’ve seen it is just through secure messaging.”	[Physician; Site A]		Clinician-patient communication
“I don’t know where to look [for notifications]. I don’t look if I haven’t received it [a notification]… Thankfully, my patients will notify me that like I was seen at whatever…they’ll often say, ‘They didn’t send you the records?’ [I reply, ] ‘No, I never [got them]’…. That’s a common story, unfortunately.”	[Physician; Site B]		Clinician-patient communication

*SEIPS *System Engineering Initiative for Patient Safety, *HIE *health information exchange, *VA *Veterans Affairs, *CPRS* Computerized Patient Record System, *JLV *Joint Longitudinal Viewer, *EHR *electronic health record

### Motivations for use, facilitators, and barriers of HIE tools

When clinicians decided to collect medication-related information from other healthcare facilities, they either used the VA HIE tools (e.g., JLV, VistA Web) or engaged in a manual process (e.g., telephone calls) to obtain the desired information. Regardless of the path taken, the clinical situations prompting their decision to seek information remained the same. *Underlying motivators* for clinicians seeking data external to their VA medical center included the desire for more comprehensive information about new patients who recently began to receive care from the clinicians’ institution, current patients concurrently or recently receiving care from an external institution, and clinicians’ concerns about possible medication discrepancies among institutions.

Clinicians’ decision about whether to use HIE tools or to gather information primarily via a manual process was influenced by facilitators and barriers they experienced, as related to the use of HIE tools. Facilitators included *familiarity* with the HIE tools and *belief* that medication-related information was *available* using an HIE tool, would be organized in the HIE in a way that was *useful*, and was *timely* for the clinical context (i.e., that clinicians believed they could find the information quickly via HIE).

Barriers to the use of HIE tools were commonly described by clinicians, and included concerns with HIE *information dependability* (e.g., incomplete or out of date information); HIE *interface challenges* (e.g., manual data entry, navigation); *lack of consistency and integration* of HIE tools (e.g., no common approach to how data elements are organized and presented to clinicians); visual clutter and *information overload (i.e.*, too much information presented from which to discern highest priority data for decision making*)*; *unfamiliarity* with the HIE tools; and lack of HIE data available from *non-VA facilities*. According to clinicians, these barriers often made the use of HIE tools too time consuming and not as worthwhile, given their competing clinical work demands.

When participants felt that the collective barriers to HIE tool use outweighed the benefits, they sometimes instead pursued a manual process to obtain external clinical data. Collectively, all clinician types described multiple strategies for manual information exchange: obtaining *direct access to non-VA EHRs* (e.g., walking across the street to an adjacent facility or logging into the other EHR); calling clinicians or pharmacies *external to their organization*; use of *physician extenders* (e.g., hiring staff to assist with medication reconciliation); and *reliance on patients’ self-reported medication information.* The interplay between the use of HIE tools vs. manual information gathering processes was influenced by factors of the *physical environment*, *organization*, and *persons*, e.g. clinician-patient and clinician-clinician communication, and medication management workflows at their respective facilities.

### Physical environment level factors

Notable influences on medication reconciliation from the physical environment pertained to *proximity* among *facilities*, such as clinicians’ ability to walk to nearby healthcare facilities to access medication-related information; *work rooms*, including distance between exam and work rooms; *printer availability*, which often limited clinicians’ printing of medication-related information; and *people* such as the clinician and patient who would ideally be well-positioned to review medication information together.

### Organization level factors

Organization-level factors influenced HIE use, including *staffing* and HIE *training*. Specifically, when facility resources enabled hiring of *dedicated medication reconciliation staff*, this was noted as a facilitator for HIE utilization. When these resources were available, support staff alleviated some time constraints during patient consultations. Finally, clinicians noted that *training* on HIE tools was often inadequate for ensuring familiarity and competency in HIE tool use.

### Person level factors

Person-level factors influencing the use of HIE tools vs. manual processes for medication reconciliation included characteristics of clinicians, their patients, clinician-clinician communication, and clinician-patient communication. Relevant clinician characteristics included their clinical *practice philosophies* and *time* available. Regarding the former, participants often preferred to focus only on medications directly related to their clinical expertise. Patient characteristics included frequent *use of both VA and non-VA clinicians for healthcare services*, *lack of patient familiarity with their medications*, and care divided across VA facilities due to *travel* (e.g., holiday or vacation travel for extended periods).

*Clinician-to-clinician communication methods* for manual HIE frequently included video chat, secure messaging, email, telephone, and electronic co-signature in the EHR. The request for co-signature generates an inbox alert to the recipient, then requiring manual confirmation of receipt that is documented in the EHR. Moreover, as noted above, clinicians commonly have *dedicated team members* responsible for medication reconciliation; these team members facilitated HIE.

With regard to *clinician-patient communication*, clinicians reported that patients were the ultimate “truth,” where any questions or uncertainty about medications defaulted to the patient’s self-report. In addition, both patients and clinicians reported a desire for improved methods for communication for external appointments to assist with accurate medication reconciliation.

## Discussion

To our knowledge, this research is the first to qualitatively describe and analyze VA clinicians’ decision-making for medication reconciliation and HIE use, while identifying underlying motivators, along with specific facilitators and barriers to the use of VA HIE tools, for medication reconciliation. The motivators, barriers, and facilitators identified in this work align with, and meaningfully add to, previous research by others, including the VHIE “superuser” survey conducted in 2018 [1, 8, 21]. Results described in our article support and expand prior literature and inform recommendations for optimizing HIE use by VA clinicians, as well as areas for future research. Ultimately, it is expected that resulting changes could improve the effectiveness of medication reconciliation activities and reduce the potential for ADEs.

First, the many *tool-related* barriers identified, such as clinicians’ concerns with the HIE interface layout and navigation options, point to opportunities to explore interface redesign for the HIE tools. While the belief that the HIE tools had accessible, useful, and timely medication-related information was a facilitator, clinicians often reported that such information was actually not available, and that design improvements are needed. Although 73% of respondents of the prior superuser survey reported daily access of HIE data, the ease of access was rated as neutral; respondents’ written comments indicated that the HIE systems were often too time consuming to use [1]. New HIE tool designs should allow for flexible navigation (e.g., sorting by specific data fields) and standardization in the data elements and layouts between HIE tools and main EHR displays of medications. In addition, information needed for medication reconciliation (e.g., starting or stopping of medications, fill history, organization where the medication was prescribed or filled) should be clearly labeled and accessible from the main HIE navigation page, with more detailed information accessible on other screens. Formal usability testing of the HIE tools should be conducted prior to and throughout the design of any new HIE interface to ensure that usability errors are identified and addressed in the redesign. Moreover, the effectiveness of new HIE interfaces in reducing usability errors and time spent on medication reconciliation should be evaluated after implementation.

Clinicians’ satisfaction and confidence in the comprehensiveness of their medication reconciliation could also be explored. Witry et al. found that non-VA clinicians caring for dual-use patients report negative experiences (e.g., inability to find pertinent information when scanned, too much information, and failure to share information with the VA) in attempting to reconcile VA and non-VA medications [22]. Therefore, future research on the impacts of HIE tool use by VA clinicians on communication inside and outside VA [23], and shared experiences pertaining to medication reconciliation, could be valuable [24].

Second, *organization-related* barriers exhibit a need for improvements in training on HIE tools. Improving the usability of HIE should be the first priority, so that it can be readily used even by trainees, novice clinicians, newcomers to the system, and clinicians who need HIE infrequently. Although training was a facilitator, clinicians were often unfamiliar with HIE tools available to them, and many reported inadequate, if any, HIE training. This aligns with findings from the superuser survey, in which 83% of clinician respondents reported being “self-trained” on HIE tools [1]. New HIE training modules should be developed and required for completion during clinician onboarding. These modules should be brief, available on demand for reference later, and focus on relevant clinical tasks, such as conducting medication reconciliation activities via HIE tools.

Third, staffing models for clinics can be optimized through identification of one or more “HIE champions” for medication reconciliation. This champion could provide support and feedback to other clinicians and field questions pertaining to the HIE tools. The use of champions, audit and feedback strategies, and ongoing education and training are well-known implementation strategies [25], and have successfully been used in the VA previously [26].

Fourth, opportunities for clinic space redesign are evident from the *physical environment-related* barriers identified. The physical placement of computer monitors, chairs, and printers could be studied through process mapping and time-and-motion analyses, building on prior work conducted in the VA setting [27–29], to specifically examine whether improvements in time spent on medication reconciliation, the accuracy of the final medication list, and patient and clinician satisfaction with the medication reconciliation process were achieved with clinic room redesign. Collectively, each of these changes could subsequently mitigate some of the *person-related* barriers identified, such as by improving workflow efficiency and increasing the amount of time clinicians have available.

While we applied SEIPS to the design of this research, it is important to note that several of our findings align with concepts from the Technology Acceptance Model (TAM) and the more recently proposed Unified Theory of Acceptance and Use of Technology (UTAUT) [30]. Both theories were developed to help explain why available technology is used or not used by the intended audience. TAM and UTAUT have been applied to IT use in healthcare. Perceived usefulness (TAM) and performance expectancy (UTAUT) refer to the belief by users that the technology will enable them to complete desired tasks. We found that that the concept of usefulness was raised both as a facilitator (e.g., clinicians would use HIE tools if they believed that information would be organized in useful ways) and as a barrier (e.g., clinicians expressed concerns with information dependability.) The importance of a user’s perceived ease of use (TAM) and effort expectancy (UTAUT) for HIE was also evident in our findings. For example, we identified interface challenges as a barrier to HIE use. Finally, our physical environment-, organization-, and person-level findings reflect the role of perceived behavioral control and facilitating conditions (UTAUT). Clinicians noted that when barriers outweighed benefits, they would simply choose not to use the HIE tools, and to seek alternatives ways of obtaining the required medication information [30].

This study has limitations. First, some of the observation opportunities were limited, as some clinicians did not have a full patient load scheduled, or patients did not keep their scheduled appointments during the study observation times. In these situations, the research team asked the clinician to talk through their processes, rather than be directly observed. We also did not quantify frequency of actual HIE use. Second, we did not identify thematic differences across clinician types in decision-making, barriers, or facilitators through our interviews and observations, although this merits further research. Prior research conducted within another VA setting has suggested differences in how pharmacists and physicians approach medication reconciliation and that these differences can influence patient health outcomes [31]. Similarly, although we aimed to recruit approximately equal numbers of inpatient and outpatient clinicians, most of the clinicians in our sample practiced in the outpatient setting or a combined inpatient and outpatient role, and thus, our findings are not as relevant to inpatient clinicians or hospitalists. Additionally, when study sites that we visited used dedicated staff for medication reconciliation, it was often a dedicated “HIE pharmacist”. Other types of research methods, such as a quantitative measure of HIE access frequency and time spent in HIE systems during patient appointments, might reveal differences in HIE use across clinician types. Third, this work was conducted in VA settings only. That said, given that non-VA institutions report similar challenges with HIE [32], we believe these results could be transferable to individuals seeking to implement or enhance the use of HIE tools for medication reconciliation in other care settings. We did not conduct member checking, whereby participants receive and confirm their own results based on the data that they individually contribute to the study [33]. Finally, we did not collect other participant-level variables which could influence technology use (e.g., extent of prior use with HIE tools or similar technologies, or extent to which participants felt that colleagues or supervisors expected them to use HIE tools) [30].

The study’s findings point to opportunities for future research. Formal usability testing or quantitative evaluation of HIE tools with distinct clinician types (roles) should be conducted to further explore any differences in decision-making and use of HIE, and how HIE interfaces can be optimized to support all types of clinicians involved in medication reconciliation. Additionally, future evaluations of medication reconciliation activities should be designed to strengthen the evidence base for the relationships among medication reconciliation, ADEs, and other clinical outcomes [34]. This can inform further enhancements to HIE tools.

## Conclusions

This study is believed to be among the first to qualitatively characterize clinicians’ decision-making for medication reconciliation and HIE use during usual clinical practice. We report motivators, barriers, and facilitators to clinicians’ use of HIE technologies. These findings corroborate and expand upon prior HIE research and identify further lines of inquiry for future studies, especially as the VA gradually shifts to a new EHR system—where some findings may differ—over the course of more than a decade. This study can inform changes in the design and use of HIE technologies, which may improve the effectiveness of clinicians’ medication reconciliation activities, and thereby reduce ADEs for patients.

## Electronic supplementary material

Below is the link to the electronic supplementary material.

Supplementary Material 1.

## Data Availability

Data is provided within the manuscript or supplementary information files.

## Abbreviations

VA: Veterans Affairs
HIE: Health informatic exchange
EHR: Electronic health record
ADE: Adverse drug events
VAMC: Veterans Affairs Medical Center
DoD: Department of Defense
VHIE: Veterans Health Information Exchange
JLV: Joint Longitudinal Viewer
SEIPS: System Engineering Initiative for Patient Safety
TAM: Technology Acceptance Model
UTAUT: Unified Theory of Acceptance and Use of Technology

## References

[1] Wing K, Bouhaddou O, Hsing N, Turvey C, Klein D, Nelson J, et al. Motivation and barriers to using the Veterans Health Information Exchange: a Survey of Veterans affairs Superusers. AMIA Annu Symp Proc. 2019;2019:913–22. 32308888 PMC7153141

[2] One Hundred Fifteenth Congress of the United States of America. VA Maintaining Internal Systems and Strengthening Integrated Outside Networks Act of 2018 (VA MISSION Act), S.2372. 2018.

[3] Linsky A, Simon SR. Medication discrepancies in integrated electronic health records. BMJ Qual Saf. 2013;22:103–9. 10.1136/bmjqs-2012-001301. 10.1136/bmjqs-2012-001301 23100547

[4] Chrischilles EA, Helling DK, Booth BM, Lemke JH, Mustion AL. Documentation and appropriateness of prescribing for Veterans Administration ambulatory-care patients. Am J Hosp Pharm. 1988;45:2345–51. 10.1093/ajhp/45.11.2345. 3067571

[5] Mello MM, Adler-Milstein J, Ding KL, Savage L. Legal barriers to the growth of Health Information Exchange-boulders or pebbles? Milbank Q. 2018;96:110–43. 10.1111/1468-0009.12313. 29504197 10.1111/1468-0009.12313 PMC5835678

[6] Devine EB, Totten AM, Gorman P, Eden KB, Kassakian S, Woods S, et al. Health Information Exchange Use (1990–2015): a systematic review. EGEMS (Wash DC). 2017;5:27. 10.5334/egems.249. 29881743 10.5334/egems.249 PMC5983051

[7] Donahue M, Bouhaddou O, Hsing N, Turner T, Crandall G, Nelson J, et al. Veterans Health Information Exchange: successes and challenges of Nationwide Interoperability. AMIA Annu Symp Proc. 2018;2018:385–94. 30815078 PMC6371252

[8] Franzosa E, Traylor M, Judon KM, Aquino VG, Schwartzkopf AL, Boockvar KS, et al. Perceptions of event notification following discharge to improve geriatric care: qualitative interviews of care team members from a 2-site cluster randomized trial. J Am Med Inf Assoc. 2021;28(8):1728–35. 10.1093/jamia/ocab074. 10.1093/jamia/ocab074 PMC8324223 33997903

[9] Byrne CM, Mercincavage LM, Bouhaddou O, Bennett JR, Pan EC, Botts NE, et al. The Department of Veterans affairs’ (VA) implementation of the virtual Lifetime Electronic Record (VLER): findings and lessons learned from Health Information Exchange at 12 sites. Int J Med Inf. 2014;83:537–47. 10.1016/j.ijmedinf.2014.04.005. 10.1016/j.ijmedinf.2014.04.005 24845146

[10] U.S. Department of Veterans Affairs. Veterans Health Information Exchange (VHIE). 2023. https://www.va.gov/VHIE/VHIE_Participating_Partners.asp. Accessed 6 Jul 2023.

[11] Hynes DM, Perrin RA, Rappaport S, Stevens JM, Demakis JG. Informatics resources to support health care quality improvement in the veterans health administration. J Am Med Inf Assoc. 2004;11:344–50. 10.1197/jamia.M1548. 10.1197/jamia.M1548 PMC516240 15187063

[12] Kolodner RM. Health Information Technology 101: Basics for Hospitals. 2004. http://www.ehcca.com/presentations/hitsummit1/1_02_2.pdf. Accessed 9 Jul 2023.

[13] Curtis AC, Gillon J, Malmrose DC. Integration of longitudinal electronic records in a large healthcare enterprise: the U.S. Veterans Health Administration experience. Stud Health Technol Inf. 2007;129:367–71. 17911741

[14] Dobre J, Carter T, Herout J, Cournoyer A. Minimizing the impact of interoperability errors on clinicians. Proc Int Symp Hum Factors Ergon Health Care. 2018;7:64–8.

[15] Department of Veterans Affairs Office of Information and Technology. Joint Legacy Viewer (JLV) 2.8.1 User Guide, Version 1.1. 2019.

[16] Holden RJ, Carayon P, Gurses AP, Hoonakker P, Hundt AS, Ozok AA, et al. SEIPS 2.0: a human factors framework for studying and improving the work of healthcare professionals and patients. Ergonomics. 2013;56:1669–86. 10.1080/00140139.2013.838643. 24088063 10.1080/00140139.2013.838643 PMC3835697

[17] Holden RJ, Carayon P. SEIPS 101 and seven simple SEIPS tools. BMJ Qual Saf. 2021;30:901–10. 10.1136/bmjqs-2020-012538. 10.1136/bmjqs-2020-012538 PMC8543199 34039748

[18] Carayon P, Schoofs Hundt A, Karsh B-T, Gurses AP, Alvarado CJ, Smith M, et al. Work system design for patient safety: the SEIPS model. Qual Saf Health Care. 2006;15(Suppl 1):i50–8. 17142610 10.1136/qshc.2005.015842 PMC2464868

[19] Bradley EH, Curry LA, Devers KJ. Qualitative data analysis for health services research: developing taxonomy, themes, and theory. Health Serv Res. 2007;42:1758–72. 17286625 10.1111/j.1475-6773.2006.00684.x PMC1955280

[20] Tong A, Sainsbury P, Craig J. Consolidated criteria for reporting qualitative research (COREQ): a 32-item checklist for interviews and focus groups. Int J Qual Health Care. 2007;19:349–57. 17872937 10.1093/intqhc/mzm042

[21] Eden KB, Totten AM, Kassakian SZ, Gorman PN, McDonagh MS, Devine B, et al. Barriers and facilitators to exchanging health information: a systematic review. Int J Med Inf. 2016;88:44–51. 10.1016/j.ijmedinf.2016.01.004. 10.1016/j.ijmedinf.2016.01.004 PMC4778080 26878761

[22] Witry M, Klein D, Alexander B, Franciscus C, Turvey C. Medication list discrepancies and therapeutic duplications among dual Use veterans. Fed Pract. 2016;33:14–20. 27840570 PMC5103625

[23] Boockvar KS, Koufacos NS, May J, Schwartzkopf AL, Guerrero VM, Judon KM, et al. Effect of Health Information Exchange Plus a Care transitions intervention on post-hospital outcomes among VA Primary Care patients: a Randomized Clinical Trial. J Gen Intern Med. 2022;37(16):4054–61. 10.1007/s11606-022-07397-5. 35199262 10.1007/s11606-022-07397-5 PMC9708976

[24] Boockvar KS, Ho W, Pruskowski J, DiPalo KE, Wong JJ, Patel J, et al. Effect of health information exchange on recognition of medication discrepancies is interrupted when data charges are introduced: results of a cluster-randomized controlled trial. J Am Inf Assoc. 2017;24(6):1095–101. 10.1093/jamia/ocx044. 10.1093/jamia/ocx044 PMC7651981 28505367

[25] Waltz TJ, Powell BJ, Matthieu MM, Damschroder LJ, Chinman MJ, Smith JL, et al. Use of concept mapping to characterize relationships among implementation strategies and assess their feasibility and importance: results from the Expert recommendations for Implementing Change (ERIC) study. Implement Sci. 2015;10:109. 10.1186/s13012-015-0295-0. 26249843 10.1186/s13012-015-0295-0 PMC4527340

[26] Sanchez SH, Sethi SS, Santos SL, Boockvar K. Implementing medication reconciliation from the planner’s perspective: a qualitative study. BMC Health Serv Res. 2014;14:290. 10.1186/1472-6963-14-290. 24996538 10.1186/1472-6963-14-290 PMC4226973

[27] Weiler DT, Satterly T, Rehman SU, Nussbaum MA, Chumbler NR, Fischer GM, et al. Ambulatory clinic exam Room Design with respect to Computing devices: a Laboratory Simulation Study. IISE Trans Occup Ergon Hum Factors. 2018;6:165–77. 10.1080/24725838.2018.1456988. 30957056 10.1080/24725838.2018.1456988 PMC6448389

[28] Saleem JJ, Weiler DT, Satterly T, Nussbaum MA, Chumbler NR, Fischer GM, et al. Field Investigation of Ambulatory Clinic Exam Room Design with respect to Computing devices: a pilot study. Proc Hum Factors Ergon Soc Annu Meet. 2018;62:518–22. 10.1177/1541931218621118. 30294199 10.1177/1541931218621118 PMC6171758

[29] Read JM, Weiler DT, Satterly T, Soares C, Saleem JJ. Provider preference in exam room Layout Design and Computing. Appl Clin Inf. 2019;10:972–80. 10.1055/s-0039-3401813 PMC6930840 31875647

[30] Holden RJ, Karsh BT. The Technology Acceptance Model: its past and its future in health care. J Biomed Inform. 2010;43:159–72. 10.1016/j.jbi.2009.07.002. 19615467 10.1016/j.jbi.2009.07.002 PMC2814963

[31] Mergenhagen KA, Blum SS, Kugler A, Livote EE, Nebeker JR, Ott MC, et al. Pharmacist- versus physician-initiated admission medication reconciliation: impact on adverse drug events. Am J Geriatr Pharmacother. 2012;10:242–50. 10.1016/j.amjopharm.2012.06.001. 22819386 10.1016/j.amjopharm.2012.06.001

[32] Everson J. The implications and impact of 3 approaches to health information exchange: community, enterprise, and vendor-mediated health information exchange. Learn Health Syst. 2017;1:e10021. 10.1002/lrh2.10021. 31245558 10.1002/lrh2.10021 PMC6508570

[33] Blandford A, Furniss D, Makri S. Ensuring quality in qualitative research. In: Blandford A, Furniss D, Makri S, editors. Qualitative HCI Research: going behind the scenes. Springer Cham; 2016. pp. 87–96.

[34] Guisado-Gil AB, Mejías-Trueba M, Alfaro-Lara ER, Sánchez-Hidalgo M, Ramírez-Duque N, Santos-Rubio MD. Impact of medication reconciliation on health outcomes: an overview of systematic reviews. Res Social Adm Pharm. 2020;16:995–1002. 10.1016/j.sapharm.2019.10.011. 31883776 10.1016/j.sapharm.2019.10.011

