# Supplementary material for: Clinicians’ use of Health Information Exchange technologies for medication reconciliation in the U.S. Department of Veterans Affairs: a qualitative analysis
Source: BMC Health Serv Res. 2024 Oct 8;24:1194. doi: 10.1186/s12913-024-11690-w (PMC11460093; doi:10.1186/s12913-024-11690-w)
Supplement: Supplementary file 1 — Supplementary Material 1. [file 12913_2024_11690_MOESM1_ESM.docx]

The interview will take approximately 30 minutes. Your participation in this interview is voluntary. You can stop the interview at any time, and let us know if you’d rather not answer a particular question. To make sure we capture all of the information you give us, we would like to audio-record this interview, but you can ask me to turn off the recorder at any time. To protect patient privacy, please do not use patients’ names or identifying information. Do you have any questions before we begin? Is it OK if I turn on the recorder now?

*Interview questions will be aimed at understanding the strengths and weaknesses of HIE design and how HIE is integrated into medication reconciliation processes. Some items of the semi-structured interviews may be adapted to become more tailored to participants' responses. The interviews will include questions such as contained in the following guide.*

**Participants may not be familiar with the term: Health Information Exchange (HIE). Consider asking about specific term or program is used at the VAMC site (e.g., VistA Web, Joint Legacy Viewer(JLV), etc).*

**Ice-Breaker Questions:**

Can you tell me a little bit about your clinical practice? (Examples of probes: in what settings [e.g., inpatient, clinics, emergency department] do you practice? For how long have you been practicing? What types of patients do you evaluate and treat?)

Health information exchange—or “HIE”—is the electronic mobilization and transmission of health information across institutions. VistA Web and the Joint Legacy Viewer (JLV) are examples of HIE tools that can access health information from other places. If you access electronic information from another healthcare facility or system, you are using health information exchange. What has been your experience with this for your patients?

**Domain: Accessing medication information with HIE**

1. In caring for Veterans, what strategies do you use to access medication information from other VA or non-VA facilities?
   1. ***What tools or systems do you use to access health information from other places? How do you decide to use these tools?**
2. What information do you access through these tools? (examples to probe: medication names, prescriber, dosage, etc.?)
3. How often do you access HIE tools?

**Domain: How HIE supports medication reconciliation**

1. Many clinicians are involved in reviewing and reconciling medications for their patients. What is your role in this process?
2. Do you use any HIE tools when you manage or reconcile medications?
3. What steps do you follow to use HIE tools to reconcile or manage medications?
   1. ***How do you use HIE tools to complete medication reconciliation?**
4. ***What information in the HIE tool or interface is helpful? Not helpful? Why?**
5. In what situations are you most likely to use HIE tools for medication reconciliation?
   1. Please explain.

**Domain: Features of HIE that support medication reconciliation**

1. In what ways does health information exchange help you with medication reconciliation?
2. What aspects of health information exchange do you find easy to use? Why?

**Domain: Barriers to using HIE for medication reconciliation**

1. In what ways does health information exchange hinder you while completing medication reconciliation? How?
2. ***What aspects of health information exchange do you find difficult to use? Why?**
3. Do you ever use paper or make handwritten notes to assist you as you use health information exchange for medication reconciliation? Why?
4. ***Can you describe a time you wanted to use health information exchange for medication reconciliation, but was unable to do so?**

**Domain: HIE Recommendations**

1. ***If you could make any changes to the *user interface* for health information exchange, what would you change about it to help with medication reconciliation?**
   1. Why would this better support medication reconciliation?
2. Are there any types of information that are unavailable to you in the HIE tool that you would like to see? Why?
3. Is any functionality missing from the HIE tool that you would like to have? Why?

**Close Out Question:**

***Is there anything about medication reconciliation or health information exchange that we did not talk about but should have?**

Thank you so much for your time in helping us to understand your perspectives about health information exchange.

*****These items have higher priority than others. Focus on these items if time is running short.
